# Modified IDSA/ATS minor criteria for severe community-acquired pneumonia best predicted mortality: Erratum

**DOI:** 10.1097/MD.0000000000006015

**Published:** 2017-01-20

In the article “Modified IDSA/ATS minor criteria for severe community-acquired pneumonia best predicted mortality”,^[1]^ which appeared in Volume 94, Issue 36 of *Medicine*, Dr. Guo's correspondence address was originally given incorrectly. The correct address is as follows:

Qi Guo, Affiliated Futian Hospital, Guangdong Medical College, Shenzhen, Guangdong 518033, China
